# Multi-level modeling of social factors and preterm delivery in Santiago de Chile

**DOI:** 10.1186/1471-2393-8-46

**Published:** 2008-10-08

**Authors:** Jay S Kaufman, Faustino T Alonso, Paulina Pino

**Affiliations:** 1 División Epidemiología, Escuela de Salud Publica, La Universidad de Chile, Santiago, Chile; 2 Department of Epidemiology, University of North Carolina, School of Public Health, Chapel Hill, NC 27599-7435, USA

## Abstract

**Background:**

Birth before the 37th week of gestation (preterm birth) is an important cause of infant and neonatal mortality, but has been little studied outside of wealthy nations. Chile is an urbanized Latin American nation classified as "middle-income" based on its annual income per capita of about $6000.

**Methods:**

We studied the relations between maternal social status and neighborhood social status on risk of preterm delivery in this setting using multilevel regression analyses of vital statistics data linked to geocoded decennial census data. The analytic data set included 56,970 births from 2004 in the metropolitan region of Santiago, which constitutes about 70% of all births in the study area and about 25% of all births in Chile that year. Dimensionality of census data was reduced using principal components analysis, with regression scoring to create a single index of community socioeconomic advantage. This was modeled along with years of maternal education in order to predict preterm birth and preterm low birthweight.

**Results:**

Births in Santiago displayed an advantaged pattern of preterm risk, with only 6.4% of births delivering before 37 weeks. Associations were observed between risk of outcomes and individual and neighborhood factors, but the magnitudes of these associations were much more modest than reported in North America.

**Conclusion:**

While several potential explanations for this relatively flat social gradient might be considered, one possibility is that Chile's egalitarian approach to universal prenatal care may have reduced social inequalities in these reproductive outcomes.

## Background

Birth before the 37th week of gestation (preterm birth) is an important cause of infant and neonatal mortality [1]. Nonetheless, the etiology of spontaneous preterm birth is still largely unknown [2]. Populations demonstrate widely varying risks of the outcome, but the specific genetic, dietary, behavioral or psychosocial factors that contribute to this variation remain obscure [3]. Researchers have described consistent socioeconomic patterns in preterm delivery risk in developed countries [4]. These factors include maternal variables such as education [5] and income [6], as well as level of neighborhood factors such as deprivation [7] and crime [8]. Despite a substantial literature on social factors and preterm birth in the US and Europe, however, there is little research reported from middle income countries. Literature searches revealed a few articles from the developing world on social factors in relation to infant mortality [9], low birthweight or other adverse reproductive events [10], but the relation between preterm delivery and social variables appears to be largely unstudied outside of wealthy societies.

Chile is a Latin American nation with just over 16 million inhabitants, about 85% of which are urbanized, with about 40% of the population living in the capital district of Santiago. It is representative of a large number of nations outside of North America and Europe that are rapidly urbanizing, integrating women into the formal economy and experiencing an epidemiologic and demographic transition away from high levels of childhood mortality and toward an older population age structure and higher burden of chronic diseases. The World Bank currently includes 96 nations as "middle income" nations – gross national income (GNI) per capita of more than $875 but less than $10,726 – including Chile, which had a 2005 GNI of about $6000 [11]. Although these 96 nations comprise about half of the world's population, epidemiology of preterm birth in relation to social factors has been studied very little to date in this social and economic context.

We therefore set out to estimate the relation between maternal social status and neighborhood social status on risk of preterm delivery in an urbanized region of a middle income country, as well as the joint relations between these factors in multilevel regression analyses. We used maternal education as an indicator of individual social status and an index created from decennial census data as an indicator of neighborhood social status. By geocoding maternal addresses from birth certificates, we were able to assign exposure values to births as recorded in vital records data.

## Methods

### Data

We studied all births in the metropolitan region of Santiago de Chile (population = 5,734,070) in the year 2004. Vital registration in Chile is virtually complete and quality of data is generally regarded as meeting high standards [12]. There were a total of 82,171 births that occurred during the study period within the 34 "comunas" that define the metropolitan region, of which 82,015 (99.8%) had complete maternal address information on the birth certificate. These births represent over a third (35.6%) of all births in the entire nation of Chile for the study year. Exclusions were made for 216 (0.3%) observations that had missing or implausible clinical estimates for gestational age at birth, and 66 (0.1%) observations that had missing or implausible birth weights. Another 82 (0.1%) were excluded because of inconsistency between the gestational age estimate and the birth weight. We also excluded 1692 (2.0%) instances of multiple births, since these births tend to be lighter and to occur earlier than singleton births.

### Exposure

In addition to individual level information from the birth record on mother's age and number of years of education, we also extracted mother's address at the time of birth, and used this to geocode each mother to one of the 348 census districts within the study area. Census districts in the metropolitan region of Santiago contain an average of about 10,000 inhabitants, and therefore are somewhat larger than those used in North America. Geocoding of address information from the birth records had not previously been attempted in this setting, and the data required extensive revision due to spelling inconsistencies in addresses, inability of the software (Arcview V.3.3) to accommodate Spanish language characters such as "ñ", and a large number of small streets and passages with similar names. After careful hand-processing of many of the records that failed in the initial automated match, we were able to achieve a match rate of just above 70%. This is lower than typically reported for cities in Europe and North America, but one of the first attempts to apply this technology in an urban developing nation context. Therefore, 23,811 (29.0%) births were not assigned to a census district, and are not analyzed further, leaving an effective analytic sample size of 56,970 births. To allay potential concerns about selection bias due to differential geo-coding success, we conducted two-sided t-tests between geocoded and non-geocoded birth records, which demonstrated no significant differences in mean birthweight, birth length, or weeks of gestational age. Age of mother was significantly different between groups, but the mean values differed by only about 4 months, suggesting minimal potential for bias. Furthermore, it is important to note that the final analysis sample of geocoded births accounts for almost a quarter (24.7%) of all births in the entire nation of Chile during the study year.

Census data from 2002 were assigned to each birth based on her census district of the mother's residence, but a data reduction strategy was required to avoid modeling a large number of highly correlated social indicators. Using a technique commonly applied to census data in the developed world [13,14], we used principal components analysis to reduce the dimensionality of the community-level social status information in the census. We selected 10 indicators based on prior substantive knowledge that they would discriminate in this socioeconomic context between advantaged and disadvantaged neighborhoods in a variety of domains. These 10 variables, all scaled to have high values representing socioeconomic advantage, were:

inverse density (i.e. number of domiciles per capita in the district)

percentage of homes connected to a sewer system

logarithm of the average total valuation per square meter

percentage of the population that does not self-identify as indigenous

percent of the population with formal schooling

percent of the population that is not currently unemployed and seeking work

percentage of the population that is classified as an owner or employer

percent of domiciles that have concrete paving

percent of domiciles that have indoor plumbing

the percent of domiciles that have indoor heating.

The average inter-item correlation for these 10 values is 0.62, with a scale reliability coefficient equal to 0.94. The first principal component, accounting for over 66% of the total variability in the items, is used as the index of socio-economic advantage. All 10 items load in the same direction on this factor, with loadings falling in a narrow range from roughly 0.25 to 0.35. The estimated score has good face validity as a measure of community SES as evidenced by a correlation of 0.47 with years of maternal education.

### Outcome

We studied preterm delivery, which was defined as singleton gestational age less than 37 weeks, as estimated clinically by the attending health care provider (usually a clinical nurse midwife). This assessment has proved more accurate than maternal reporting of last menstrual period alone, since it also may involve information from ultrasound [15], which is available for a majority of the births due to Chile's aggressive prenatal care program [16]. We also defined a secondary outcome of preterm low birthweight, which is a birth with gestational age of less than 37 weeks weighing less than 2500 grams [17].

### Statistical Analysis

Because of exposure information at the individual and the community level, the data have a multilevel structure. We therefore employed random effects logistic regression, allowing a district-specific intercept term to be estimated as a draw from a random distribution of intercepts, conditional on modeled covariates [18]. Exponentiated exposure coefficients therefore have an odds ratio interpretation as district-specific effects, and they approximate risk ratios due to the relatively rare outcomes studied. The exposure effect estimates also have an approximate marginal (i.e., population average) interpretation because of the low interclass correlation coefficient (ICC) for the outcomes studied (i.e., ICC < 0.05). Models were estimated using adaptive Gauss-Hermite quadrature, as implemented in Stata Version 9 [19].

## Results

Births in Santiago during the study period demonstrate a relatively advantaged pattern of outcomes, with modest risks of low birth weight (4.9%) and preterm delivery (6.4%) in comparison with developed countries (Table 1). For example, The United States prevalences of low birth weight and preterm for singleton infants in 2004 were 8.1% and 10.8%, respectively [20]. The data also demonstrate a high prevalence of births to unmarried women and generally high levels of maternal education. On the other hand, census variables depict a population with modest material levels of existence. For example, the average census district has only a quarter of homes connected to the sewer system. Nonetheless, almost all births were attended by medical personal (physicians or clinical nurse midwives).

**Table 1:** Description of variables in analytic sample, Santiago, Chile, 2004

**Continuous Birth Outcome Variable**	**N**	**Mean**	**SD**	**Min**	**Max**
Weeks of gestational age at birth	56868	38.62	1.80	20	43
Weight at birth in grams	56900	3344.96	529.01	508	6060
Length at birth in cm	56873	49.54	2.51	30	61
Maternal Age at birth (years)	56965	27.86	6.68	11	51
Maternal Years of Education	56966	12.31	3.37	0	22

					

**Continuous Census District Variable**	**N**	**Mean**	**SD**	**Min**	**Max**

Number of domiciles per capita	56963	0.27	0.06	0.09	0.73
% domiciles connected to sewer	56963	25.17	3.92	8.03	50.16
ln(average total valuation/m^2^)	56900	10.78	0.88	8.16	14.88
% population non-indigenous	56963	96.65	1.58	90.71	99.32
% population with formal schooling	56963	98.64	0.67	95.99	99.88
% not unemployed and seeking work	56963	95.42	0.99	92.36	99.02
% population owners or employers	56963	1.91	1.19	0.15	9.78
% domiciles with concrete paving	56963	2.19	4.94	0.00	30.43
% domiciles with indoor plumbing	56963	25.20	3.91	8.03	50.16
% domiciles with indoor heating.	56963	21.22	4.84	6.52	36.03
					
Census District SES Score^1^	56894	0.00	1.00	-2.15	4.70

					
	
	**In Full Sample**			**Ages 20+ Only**^2^	

**Categorical Birth Outcome Variable**	**N**	**%**		**N**	**%**

Male Sex of Newborn	56970	51.05		49771	51.19
Medical Attention at Birth	56970	99.96		49771	99.96
Mother Married at time of Birth	56970	53.31		49771	47.60
Preterm Deliver (< 37 wks)	56868	6.41		49674	6.29
Low Birth Weight (< 2500 g)	56900	4.94		49707	4.80
Preterm LBW (< 2500 g, <37 wks)	56802	3.33		49613	3.27
Very Low Birth Weight (< 1500 g)	56900	0.86		49707	0.86

1 based on principal components analysis of 10 census items

**Figure 1:**
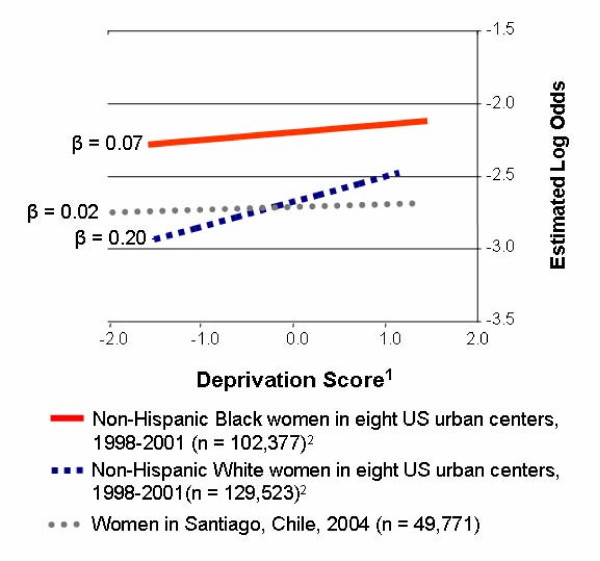
**Estimated log odds of preterm delivery (adjusted for maternal age and education, women ages 20+); Chile and US**. ^1 ^Positive numbers indicate greater community-level material deprivation, with comparison at equal points in the distribution of scores for each country. ^2 ^From reference 7 (O'Campo et al, 2008).

2 for comparison with Figure 1

A relationship between individual maternal education and preterm delivery is observed in the data, but it is modest in magnitude (Table 2). When adjusted for maternal age, sex of child and parity, women with less than 12 years of education have a nearly 20% excess odds of preterm delivery and a nearly 30% excess odds of preterm low birthweight delivery, compared to women with more than a high school education. This analysis shown in Table 2 is restricted to those 18 years of age and older because completed years of education is undefined for younger women. The neighborhood SES variable is also a modest predictor of preterm birth, both crudely and after adjustment for maternal age, sex of child, parity and maternal education. Results are shown in Table 3 for both the continuous score (scaled so that 1 unit is a standard deviation) and in quartiles with the highest SES group as the referent. No obvious dose-response relation is evident for SES score in relation to risk, although all groups are estimated to have elevated odds of the outcomes in relation to the most advantaged SES quartile. Magnitude of effect for individual maternal education does not change substantially after adjustment for neighborhood SES in these models, maintaining a 20–25% excess odds of the outcomes (data not shown). Tests for adding quadratic terms for the continuous SES measure are not significant, and likelihood ratio tests for intraclass correlation coefficients > 0 are significant in all models, indicating that there is detectable clustering by census district. Nonetheless, the magnitudes of these estimated intraclass correlation coefficients are small (< 0.01) and so estimates also maintain an approximate marginal interpretation.

**Table 2:** Association between years of maternal education and preterm delivery among mothers 18 years of age and older, Santiago, Chile, 2004

Maternal Education (years)	Preterm Delivery		Preterm Low Birthweight Delivery	
	Crude	Adjusted^3^	Crude	Adjusted^3^
	
	OR (95% CI)	OR (95% CI)	OR (95% CI)	OR (95% CI)
< 12 yrs	1.12 (1.03, 1.23)	1.17 (1.06, 1.29)	1.14 (1.01, 1.29)	1.27 (1.11, 1.45)
= 12 yrs	1.05 (0.97, 1.14)	1.10 (1.01, 1.20)	1.12 (1.00, 1.26)	1.20 (1.06, 1.35)
> 12 yrs	REFERENT	REFERENT	REFERENT	REFERENT

homogenetity^1^	p = 0.04	p < 0.01	p = 0.06	p < 0.01
trend^2^	p = 0.01	p < 0.01	p = 0.03	p < 0.01

1 test of homogeneity (equal odds)

2 test for trend of odds

3 adjusted for parity, sex of child and maternal age using logistic regression

**Table 3:** Association between neighborhood socioeconomic status^1 ^and preterm delivery among births in Santiago, Chile, 2004

Census District SES Score	Preterm Delivery		Preterm Low Birthweight Delivery	
	Crude	Adjusted^1^	Crude	Adjusted^2^
	
	OR (95% CI)	OR (95% CI)	OR (95% CI)	OR (95% CI)
1^st ^Quartile	REFERENT	REFERENT	REFERENT	REFERENT
2^nd ^Quartile	1.12 (1.00, 1.24)	1.10 (0.98, 1.23)	1.14 (0.99, 1.31)	1.13 (0.98, 1.31)
3^rd ^Quartile	1.12 (1.00, 1.24)	1.09 (0.97, 1.21)	1.19 (1.03, 1.37)	1.16 (1.00, 1.34)
4^th ^Quartile	1.07 (0.96, 1.19)	1.03 (0.92, 1.15)	1.14 (0.99, 1.31)	1.11 (0.95, 1.29)

Continuous Score	1.03 (0.99, 1.07)	1.02 (0.97, 1.06)	1.05 (1.00, 1.11)	1.04 (0.99, 1.10)

1 based on principal components analysis of 10 census items

2 adjusted for parity, sex of child, maternal age and maternal education using multi-level logistic regression with random effects for census district intercepts

## Discussion

Our study of individual education and community SES in relation to preterm birth in a middle-income country demonstrated detectable effects that were nevertheless much more modest in magnitude than what is commonly observed in the US and Europe. Figure 1 compares data from the current study with pooled data from eight urban centers in the US on a standardized axis for the exposure [7]. Data from Chile have been restricted to n = 49,771 women aged 20 years and older for great comparability with the US data. It is important to note that the absolute level of economic development at a specific value for this index is not comparable between Chile and the US, but rather this is a comparison at equal points in the distribution of scores in each country. If one views the African-American population as an "under-developed" population within the US (in the sense that as a group there has been less investment in human capital and neighborhood infrastructure), then this graph suggests a continuum of economic levels in which neighborhood SES would become increasingly important as the average level of development of the population increases. This represents an intriguing hypothesis that could be investigated in subsequent studies in a greater variety of economic contexts. Furthermore, this comparison shows that although the Non-Hispanic White US women studied have a greater slope of neighborhood deprivation, their average risk of preterm delivery is roughly comparable to that observed in Santiago. These US women therefore have considerably greater heterogeneity in risk by neighborhood than the women from Chile.

These results are also consistent with the pattern of adverse outcomes observed in previous studies for Hispanic migrants in the US [21,22]. Risk of adverse birth outcomes is consistently seen to be modest in Hispanic US women, a phenomenon often referred to as the "Hispanic Paradox" [23]. Moreover, Hispanic women in the US have much more modest SES gradients of risk than do women who identify as Non-Hispanic White and Non-Hispanic Black. This is especially true for foreign-born Hispanic women [22]. The current analysis shows that for preterm delivery, the pattern among women in Santiago, Chile is indistinguishable from that seen for these foreign-born US Hispanic women in this regard, which is perhaps not unsurprising. Most previous literature on this topic considers gradients in risk associated with educational attainment of the mother, since this is a variable widely available in US vital statistics data. The present study shows that this applies equally well to neighborhood deprivation, as measured by census data. Although few published studies have considered risk of preterm delivery for US Hispanic women by neighborhood socioeconomic characteristics, Masi et al. similarly observed no important influence of neighborhood economic characteristics on preterm delivery for Hispanic women in Chicago [24].

The surprisingly healthy profile of birth risk in Chile compared to the US, despite being a much poorer country, may reflect substantial differences in the organization of medical services for pregnant woman. The current program for universal provision of prenatal care was developed by the Ministry of Health in the late 1970s. There are no official statistics for the actual coverage of this ostensibly obligatory prenatal care, due to the fact that many women who qualify for public health insurance (FONASA) choose to instead attend private health care providers during pregnancy. In 1983 an early estimate of national coverage showed that 91% of pregnant women had prenatal care throughout pregnancy [25]. A later study conducted in the largest public hospital in Santiago in 1991–1992 showed that only 1.2% of the deliveries failed to receive required prenatal care control [26]. More contemporary analyses based on 2005 data from FONASA and the Health Statistics and Information Department of the Ministry of Health shows that the prenatal care program has a coverage of at least 98% of the population that is affiliated to FONASA in the Metropolitan Region around Santiago. Moreover, nearly 78% of those women are estimated to initiate prenatal control by a clinical provider before the end of the first trimester [27,28].

The Chilean prenatal care program prides itself on an integrated multidisciplinary perspective with the intention of providing for complete physical and mental health of the mother during pregnancy and the mother-child pair after parturition. The program includes at least 6 professional visits by clinical nurse midwifes and/or physicians, referral to tertiary health care facilities if needed, educational sessions, laboratory exams including blood and urine monitoring (e.g., glucose blood level) as well as glucose tolerance testing, and screening for HIV, syphilis, Chagas disease, and cervical cancer. Women also receive three ultrasound examinations around gestational weeks 14, 24 and 34. Dietary supplements are also included and distributed according the nutritional status of the mother [29]. All of these services are intended to be universal, available locally via a practitioner who has an ongoing relationship to the pregnant woman, and completely covered by universal public health insurance (FONASA). While severe social inequalities exist in many other sectors of the Chilean health system [30], services for pregnant women are intended to be completely free of access barriers due to cost, travel distance or bureaucratic hurdles. While this goal may not always be achieved, it seems that the structure of the system is inherently more egalitarian in intent and more broad in scope than many of its North American counterparts. Recent randomized intervention studies in the US support the hypothesis that integrated prenatal care services can have a substantial beneficial effect on pregnancy outcomes [31].

The observed modest socioeconomic gradient in preterm birth in these data does not imply socioeconomic equality in health more generally. Chile remains a profoundly unequal society in many respects, with one of the highest gini coefficients in the world for income inequality (56.5, compared to 40.8 in the US) [[32], p. 332]. Other health outcomes appear to reflect these inequalities more directly, again pointing to the somewhat unique attention to healthy pregnancy as a long-standing Chilean policy priority. For example, deaths of infants in the first year of life in Chile fell from 17.0 per 1000 live births in 1990 to 9.4 per 1000 live births in 2000 [33]. Despite the dramatic overall improvement, however, inequalities grew more severe in the social distribution of the remaining deaths. In 2003 data, for example, infant mortality was 29.7 per 1000 live births among uneducated mothers compared with only 5.3 per 1000 among those with 13 or more years of education [34]. The overall improvements in infant mortality in the decade following democratization were not accompanied by improvements in the already impressively low risks of preterm birth or low birthweight [33], however, suggesting that these mortality gains for newborns were produced by the introduction of improved medical services for infants rather than by improvements in more fundamental social causes of adverse outcomes such as poverty and malnutrition [35].

While these data appear consistent with a more modest effect of social factors on preterm birth at the individual and community level, there are potential alternate explanations. One might question data quality, but this seems the least likely explanation for the findings, given the consistently observed advantage for Chilean pregnancies and the resources and attention devoted to data integrity in Chile. A more plausible concern is that our a priori decisions about relevant census variables may have failed to capture true social variation between communities [36]. For example, census districts are not salient community divisions for women living in Santiago, and their boundaries are often arbitrary with respect to socially recognized neighborhoods [37]. We also fail to account for many other plausibly important psychosocially mediated exposures, including family and social support during pregnancy, diet, exercise and occupational stress [38]. Finally, the relatively low proportion of successfully geocoded births could conceivably obscure a more marked socioeconomic pattern in the complete population than is observed in the analytic data set. More comprehensive exposure assessment in middle-income nations will fill important gaps in our nascent understanding of reproductive health [39].

## Conclusion

We conducted a study of maternal social status and neighborhood social status on risk of preterm delivery in a middle-income country, using vital statistics and geocoded decennial census data. Analysis of 56,970 births that occurred in 2004 in the metropolitan region of Santiago revealed a relatively advantaged pattern of preterm risk overall, with only 6.4% of births delivering before 37 weeks. Moreover, although associations were observed between risk of outcomes and individual and neighborhood factors, the magnitudes of these associations were much more modest than those reported in North America. This relatively flat socioeconomic gradient for adverse birth outcomes is similar, however, to that reported for Hispanic women in the United States, especially those that are born in other countries. We considered several potential explanations for the modest social gradient in risk observed in these data, including the potential impact of Chile's relatively egalitarian approach to universal prenatal care.

## Competing interests

The authors declare that they have no competing interests.

## Authors' contributions

JSK conceptualized and led the study, conducted the statistical analysis and led the writing of the manuscript. FTA assembled and managed the data sets, geocoded the data, assisted in analysis and interpretation, and collaborated in the writing of the paper. PP obtained the raw data, organized the research effort, consulted on all substantive and analytic questions, and collaborated in the writing of the paper.

## Pre-publication history

The pre-publication history for this paper can be accessed here:


